# Heat-Priming during Somatic Embryogenesis Increased Resilience to Drought Stress in the Generated Maritime Pine (*Pinus pinaster*) Plants

**DOI:** 10.3390/ijms24119299

**Published:** 2023-05-26

**Authors:** María Amparo Pérez-Oliver, María del Carmen González-Mas, Begoña Renau-Morata, Isabel Arrillaga, Ester Sales

**Affiliations:** 1 Biotechnology and Biomedicine (BiotecMed) Institute and Plant Biology Department, Faculty of Pharmacy, Universitat de València, Vicent Andrés Estellés s/n, Burjassot, 46100 Valencia, Spain; maria.a.perez-oliver@uv.es (M.A.P.-O.); carmen.gonzalez-mas@uv.es (M.d.C.G.-M.); begonya.renau@uv.es (B.R.-M.); 2 Agrarian and Environmental Sciences Department, Institute of Environmental Sciences (IUCA), University of Zaragoza, High Polytechnic School, Ctra. Cuarte s/n, 22197 Huesca, Spain; esalesc@unizar.es

**Keywords:** abiotic stress, climate change, conifer, epigenetics, forests, resilience

## Abstract

Drought stress is becoming the most important factor of global warming in forests, hampering the production of reproductive material with improved resilience. Previously, we reported that heat-priming maritime pine (*Pinus pinaster*) megagametophytes during SE produced epigenetic changes that generated plants better adapted to subsequent heat stress. In this work, we tested, in an experiment performed under greenhouse conditions, whether heat-priming will produce cross-tolerance to mild drought stress (30 days) in 3-year-old priming-derived plants. We found that they maintain constitutive physiological differences as compared to controls, such as higher proline, abscisic acid, starch, and reduced glutathione and total protein contents, as well as higher ΦPSII yield. Primed plants also displayed a constitutive upregulation of the *WRKY* transcription factor and the Responsive to Dehydration 22 (*RD22*) genes, as well as of those coding for antioxidant enzymes (*APX, SOD,* and *GST*) and for proteins that avoid cell damage (*HSP70* and *DHNs*). Furthermore, osmoprotectants as total soluble sugars and proteins were early accumulated in primed plants during the stress. Prolongated water withdrawal increased ABA accumulation and negatively affected photosynthesis in all plants but primed-derived plants recovered faster than controls. We concluded that high temperature pulses during somatic embryogenesis resulted in transcriptomic and physiological changes in maritime pine plants that can increase their resilience to drought stress, since heat-primed plants exhibit permanent activation of mechanisms for cell protection and overexpression of stress pathways that pre-adapt them to respond more efficiently to soil water deficit.

## 1. Introduction

Climate change is expected to affect forest landscape dynamics in many ways, but it is possible that the most important direct impact of global warming will be drought stress [1]. Both the frequency and intensity of drought events will increase, and these adverse conditions particularly affect long-lived plant species, such as forest trees. Drought menaces tree performance both directly, by constraining photosynthesis and hydraulic conductivity in plants, and indirectly, by modifying the life cycles of biotic factors [2]. In this context, forest decline is reported worldwide, but Southern Europe is an especially vulnerable region. Mediterranean climate conditions are characterized by cold and wet winters and by a marked rainfall deficit in summer, when temperatures rise. More than 35 million ha in this area are covered by forests, with the trees’ active growing season limited to the humid period between fall and spring. Climate change projections for the Mediterranean basin are a pronounced decrease in precipitation and a significant warming (precipitation decrease exceeding 25–30% and warming exceeding 4–5 °C), especially during summer [3], resulting in soil moisture deficit, which, along with rising air temperatures and evapotranspiration, induces agricultural drought stress [4].

It is important to note that besides the tree-level effects, global warming causes a major disruption of Mediterranean stands, increasing soil erosion, the frequency and intensity of forest fires, and pest outbreaks, all threatening the continued supply of forest ecosystem services in Southern Europe [5].

Conifers dominate global arid, semi-arid, montane, and circumpolar zones, but the genomics of its drought and thermal tolerance have received little attention and lag behind studies of other plant species [6]. Two mechanisms have been described for drought adaptation in these species: an ancestral mechanism relies on high levels of abscisic acid (ABA) to close stomata and to maintain water potential, whereas the other implies leaf desiccation, lowing water potential as a signal to close stomata during sustained water stress [6,7]. Recently, strategies for engineering drought resistance in forest trees using transgenic approaches have been reviewed [8], while potential solutions based on plant immune systems, including plant stress memory, cross-stress tolerance, and seed priming have also emerged as efficient and favorable approaches for enhancing drought tolerance without employing genetic engineering technologies [9]. Priming or stress memory is the short-term acclimation response of plants to reiterated stress, meaning that plants then become more tolerant to future exposures of the same or different (cross-tolerance) stress factor. Heat or cold priming-induced cross-tolerance is very common in plants, and often results from the synergistic co-activation of multiple stress signaling pathways, which involves reactive nitrogen species (RNS), reactive oxygen species (ROS), reactive carbonyl species (RCS), plant hormones, and transcription factors [10]. Epigenetic plasticity is particularly important for long-lived species such as forest trees, and occasionally, stress memory may also be extended into future generations [11]; epigenetic-based approaches for forests-tree breeding have been reviewed [12].

Among the conifers commonly found in the Mediterranean forests, maritime pine *(Pinus pinaster* Aiton) is a fast-growing, medium-sized tree native to Southwestern Europe. Since it is cultivated for producing wood and resin, this plant species is also now found in other European regions and in North Africa, in altitudes from sea level to 2000 m, therefore distributed in a wide ecological range. Maritime pine displays large genetic variation in adaptive traits, such as drought and frost tolerance, and insect and nematode resistance, which have been observed between provenances [13,14,15]. Populations from dry climates have been reported to be adapted to drought through larger biomass allocation to roots [16] and higher water-use efficiency with lower dependence on stomata closure regulation than populations from mild climates [17]. The genetic basis of this variation in the maritime pine response to abiotic stress was analyzed by transcriptome sequencing, leading to the identification of organ-specific genes constitutively expressed as well as differentially expressed, when comparing control versus water stress conditions in drought-sensitive and drought-tolerant genotypes [18,19,20]. A recent study reveals new insights into the metabolic changes under heat and drought stress, enlightening the role of secondary metabolites in the response of maritime pine to combined abiotic stress [21].

Despite these adaptive traits, *P. pinaster* decline has recently been observed in different areas from the Iberian Peninsula. Factors such as biotic agents, water stress, and land-use legacies have been reported as the major drivers behind this tree decline [2]. Furthermore, chronic lower growth and higher dieback rates in *P. pinaster* stands have been linked to drought events [22], and cumulative impacts of stress events on tree growth and vigor have been related to higher vulnerability to drought [23]. To promote the long-term continuity of South European forests in the climate change-driven scenario, it is recommended to select reproductive material with improved resilience [5]. For this purpose, *P. pinaster* infra-specific variability can be exploited by identifying and cloning superior genotypes; although, conventional breeding programs face several constraints such as the long generation time, the polygenic regulation of many adaptive and growth traits, and the large and complex genome of this plant species [13,24]. According to [8], osmotic adjustment, antioxidative defense, and increased water-use efficiency are traits that will be important targets for enhanced drought tolerance at the cellular and tissue level.

Epigenetic-based strategies offer an interesting alternative for the improvement of drought tolerance in maritime pine. Somatic embryogenesis is the selected biotechnological tool for in vitro propagation of conifers and has been the basis for inducing epigenetic adaptations in *Abies* [25] as well as for increasing resilience to abiotic stress in *Pinus* species [26,27,28,29,30,31]. These studies reported changes in DNA methylation and differential expression of stress-related genes, as well as in tissue ultrastructure and metabolite profiles of embryogenic cells. Furthermore, *Pinus radiata* plants derived from primed somatic embryos showed adaptive traits in response to heat and drought stresses [29,31]. Factors regulating somatic embryogenesis in maritime pine using megagametophytes as initial explants have been studied by our group [32]. We have also shown that altering temperature to either colder (18 °C) or warmer (28 °C) conditions during maritime pine somatic embryo maturation resulted in plants with altered responses to heat stress [33]. In other experiments, we produced plants from heat-primed megagametophytes and demonstrated that those generated after 37 °C or 50 °C pulses during somatic embryogenesis induction showed better performance under further heat stress [30], but no effects of priming in drought stress have been described so far for the species. The objective of this work is to demonstrate whether cross-tolerance of these priming-derived plants to drought stress can be induced in maritime pine in terms of physiological measurements and gene expression analyses.

## 2. Results

Three-year-old plants derived from primed megagametophytes growing in the greenhouse, control (NP), and primed (P37 and P50) plants were used in a 30-day drought experiment. Needles from the three groups of plants were sampled before (T0), after 15 days without watering (T15), at the end of the stress period (T30), and 10 days after recovery of normal watering (TR). Irrespective of the origin (primed or not) all plants survive to water withdrawal; although, some epinasty symptoms were observed in basal needles. We analyzed the impacts of water scarcity on the osmotic adjustment, the photosynthetic status, and the carbohydrate and protein metabolism, as well as the ABA and reduced glutathione content of the maritime pine plants. We also analyzed the expression of 12 genes related to the drought stress response of this plant species.

### 2.1. Osmotic Adjustment in Maritime Pine Plants under Drought Stress

**Figure 1:**
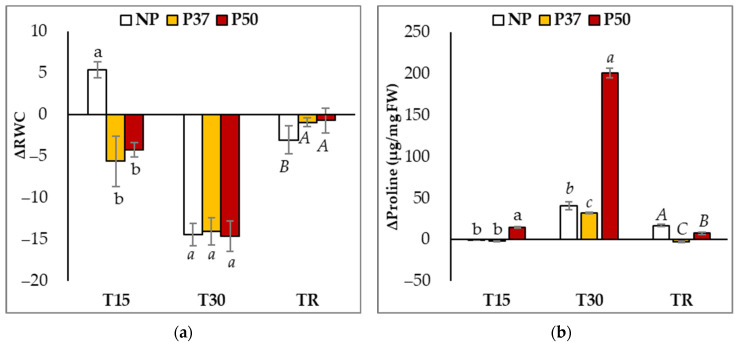
Variation in Relative Water Content (**a**) and in proline content (**b**) in needles of 3-year-old maritime pine plants generated by somatic embryogenesis from not-primed (NP) or primed at 37 °C (P37) or at 50 °C (P50) megagametophytes, when subjected to a 30-day hydric stress treatment and allowed to recover for further 10 days. Data are mean ± SE of differences referred to the mean initial content determined in needles sampled during (T15), at the end of the treatment (T30), and after the recovery period (TR), in 6 replicates each. At each sampling time, values following the same letter were not significantly different according to Tukey-b test (α = 0.05).

Irrespective of the group of plants (primed or not), the relative water content (RWC) of maritime pine needles was significantly affected by hydric stress conditions (*p* < 0.001), but the pattern of variation differed among groups (Figure 1a). After 15 days without watering, the RWC of needles was significantly reduced only in those of primed plants (5.6 and 4.2% in P37 and P50 plants, respectively) while it did not change in needles from NP plants (*p* = 0.001). At T30, however, RWC decreased similarly in the three groups of plants (on average 14.3%). Finally, differences were also observed at TR, when RWC was restored in P37 and P50 primed plants but not in NP plants, in which RWC rates were lower than those determined initially (77.2% ± 4.1 front to 80.2% ± 2.0).

Priming with high temperatures induced constitutive changes in maritime pine plants, since proline basal contents determined in needles (T0 samples) were significantly higher in those from P37 plants (*p* = 0.001) than in NP and P50 plants (22.0 ± 1.6 μg/mg FW front to 18.1 ± 2.4 and 14.6 ± 2.9 μg/mg FW, respectively; Supplementary Table S1). The proline content of maritime pine needles was also significantly affected by drought (*p* < 0.001), and the observed variation pattern along the experiment (Figure 1b) also differed among groups: basal levels remained unchanged after 15 days without watering in NP and P37 plants, while they increased in P50 plants. Higher proline contents were found at the end of the drought period (T30 samples), when these rates increased 3.6-fold in NP plants, 2.5-fold in P37 plants, and a drastic 14.8-fold in P50 plants. After the 10-day recovery time, proline contents were restored in primed plants but not in NP plants (Figure 1b). Furthermore, significantly higher (*p* < 0.001) levels of this amino acid (34.8 ± 4.1 μg/mg FW) were determined in this group after the drought experiment, as compared to those in P37 (19.4 ± 1.6 μg/mg FW) and P50 plants (22.1 ± 3.7 μg/mg FW).

### 2.2. Photosynthesis Parameters during Hydric Stress

**Figure 2:**
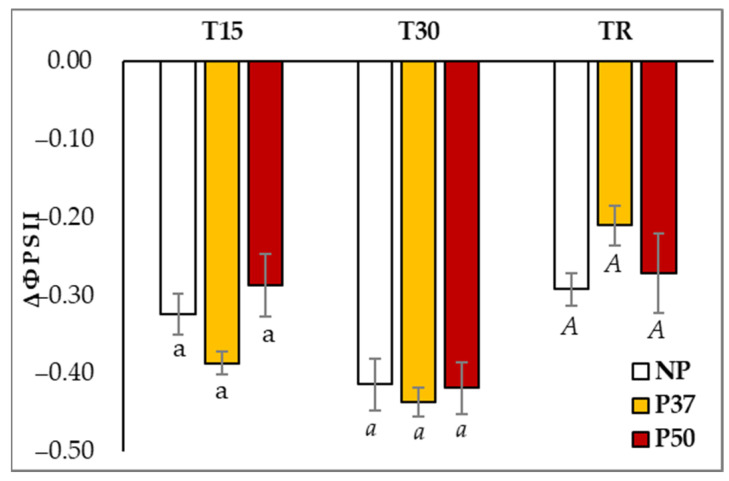
Variation in the photosynthetic activity, determined as photosystem II yield (ΦPSII), in needles of 3-year-old maritime pine plants generated by somatic embryogenesis from not-primed (NP) or primed at 37 °C (P37) or at 50 °C (P50) megagametophytes, when subjected to a 30-day hydric stress treatment and allowed to recover for further 10 days. Data are mean ± SE of ratios referred to the initial contents determined in needles sampled during (T15), at the end of the treatment (T30), and after the recovery period (TR), in 10 replicates. For each sampling time, values following the same letter were not significantly different according to Tukey-b test (*p* < 0.001).

Priming with high temperatures during somatic embryogenesis resulted in maritime pine plants with altered photosynthetic status. Basal levels of photosystem II yield (ΦPSII) were significantly higher in P37 plants as compared to those of NP and P50 plants (0.712 ± 0.013 front to 0.681 ± 0.016 and 0.678 ± 0.014, respectively; *p* = 0.014; Supplementary Table S1). However, similar patterns of variation in ΦPSII were observed in maritime pine plants from the three groups when subjected to hydric stress for 30 days (Figure 2). Irrespective of the group of plants, ΦPSII was significantly reduced along the experiment (*p* < 0.001), and photosynthesis levels were not resumed after a recovery time of 10 days; although better recovery in P37 plants was observed (*p* > 0.05).

Regarding the pigment content of needles from the maritime pine plants, no significant differences in either chlorophyll a or chlorophyll b contents (Supplementary Figure S1a) were observed in those sampled at T0 in control or primed plants (*p* = 0.169 and *p* = 0.474, respectively). Furthermore, the content in these pigments did not vary along the drought experiment (*p* = 0.474, *p* = 0.393, respectively). Similarly, the carotenoid content of maritime pine needles (Supplementary Figure S1b) did not vary among the three groups of plants (*p* = 0.327) nor along the performed experiment (*p* = 0.084). However, the carotenoid content of NP plants increased 1.4-fold after the drought period (T30), while this effect was not observed in primed plants.

### 2.3. Carbohydrates and Protein Contents of Maritime Pine Plants

**Figure 3:**
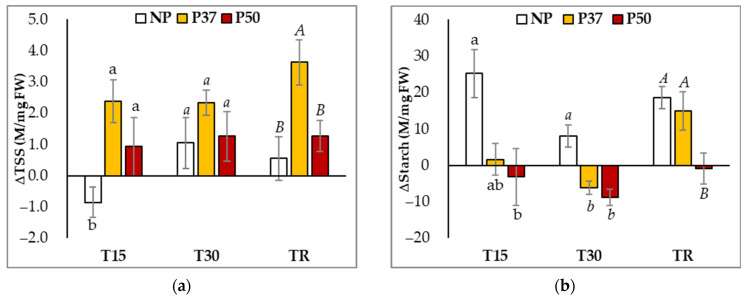
Variation in Total Soluble Sugars (**a**) and starch content (**b**) of needles of 3-year-old maritime pine plants generated by somatic embryogenesis from not-primed (NP) or primed at 37 °C (P37) or at 50 °C (P50) megagametophytes, when subjected to a 30-day hydric stress treatment and allowed to recover for further 10 days. Data are mean ± SE of differences referred to the mean initial content determined in needles sampled during (T15), at the end of the treatment (T30), and after the recovery period (TR), in 6 replicates. For each sampling time, values following the same letter were not significantly different according to Tukey-b test (α = 0.05).

Similar basal levels of total soluble sugars (TSS) were determined at T0 in needles of not-primed (NP) and primed (P37 and P50) maritime pine plants (Supplementary Table S1). Hydric stress significantly affected the TSS of needles (*p* = 0.025), since average TSS increased from 7.4 M/mg FW up to 9.3 M/mg FW. This variation was mainly explained by the drastic increase observed (Figure 3a) in total sugar content of needles sampled from P37 plants (from 6.5 ± 0.7 to 10.2 ± 0.7 M/mg FW).

In contrast, heat priming of maritime pine megagametophytes during somatic embryogenesis resulted in plants with altered starch content. Primed plants (P37 and P50) showed significantly higher constitutive starch levels (at T0) than NP plants (49.4 ± 2.5 and 49.5 ± 2.5 front to 32.8 ± 4.2 M/mg FW, respectively; *p* = 0.005). Hydric stress also significantly affected the starch content of maritime pine needles (*p* = 0.004), and the response varied among the three groups of plants (Figure 3b). Applying hydric stress induced a drastic increase in the starch content of NP plant needles, while this effect was not observed in P37 and P50 plants (Figure 3b). At the end of the drought experiment (T30), starch levels decreased in primed plants, while increasing in NP plants. When water supply was restored, at TR, starch content in needles of P50 plants recovered initial values, while in NP and P37 plants remained significantly higher than basal levels.

**Figure 4:**
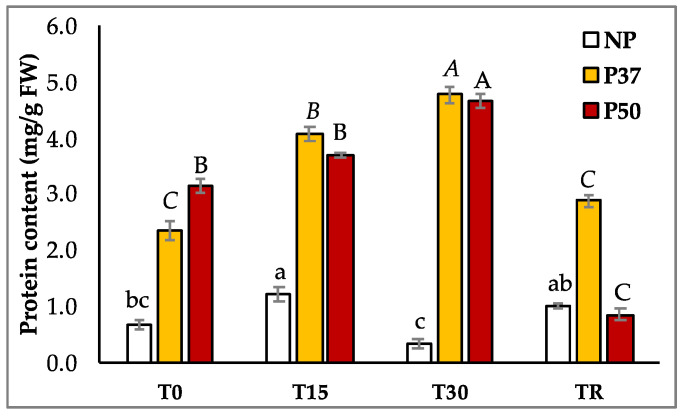
Protein content in needles of 3-year-old maritime pine plants generated by somatic embryogenesis from not-primed (NP) or primed at 37 °C (P37) or at 50 °C (P50) megagametophytes, when subjected to a 30-day hydric stress treatment and allowed to recover for further 10 days. Data are mean ± SE of 3 replicates sampled before (T0) during (T15), at the end of the treatment (T30), and after the recovery period (TR), in 3 replicates. For each group of plants, values following the same letter were not significantly different according to Tukey test (α = 0.05).

Regarding the protein content of maritime pine needles, we found significantly higher (*p* < 0.001) levels in primed plants when compared to control plants (Figure 4; Supplementary Table S1). In fact, basal (at T0) protein content in P37 plants was 3.5-fold, and in P50 plants was 4.7-fold that of NP plants (Supplementary Table S1). When plants were subjected to hydric stress, protein content varied significantly (*p* = 0.031), and the pattern of variation depended on the group of plants. In NP plants, protein content increased significantly in T15 plants but initial levels were resumed at the end of the drought period (T30) and after the recovery period (TR). In primed P37 plants, significantly higher protein contents were maintained during the entire hydric stress period and initial levels were resumed in TR samples. Finally, in P50 plants, the initial protein content did not increase after 15 days without watering, while increased significantly at the end of the drought period, in T30 samples. In contrast to results observed for NP and P37 plants, after the recovery period, the protein content of needles from P50 plants was significantly reduced as compared to levels determined initially (Figure 4). Therefore, after the drought experiment (TR), the protein contents of NP and P37 plants were not significantly different from the initial rates, being higher in P37 plants, while higher total protein contents decreased in P50 plants.

### 2.4. ABA Content in Maritime Pine Needles

**Figure 5:**
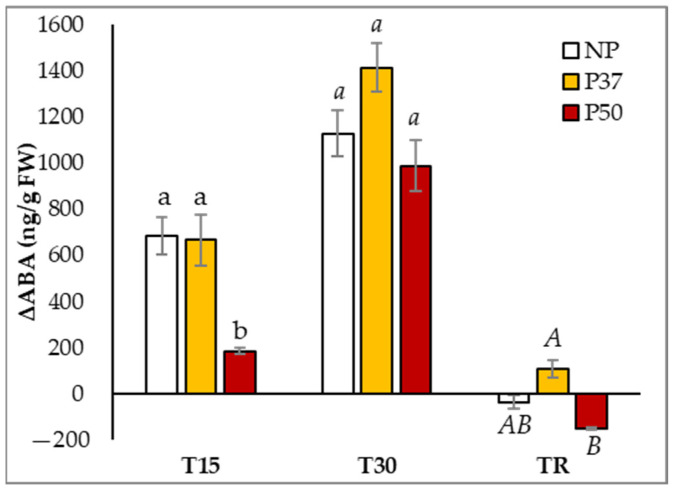
Variation in ABA contents determined in needles of 3-year-old maritime pine plants generated by somatic embryogenesis from not-primed (NP) or primed at 37 °C (P37) or at 50 °C (P50) megagametophytes, when subjected to a 30-day hydric stress experiment. Data are mean ± SE of differences referred to mean initial ABA content, of needles taken during (T15), at the end (T30) of the experiment, and after a recovery period of 10 days (TR), with 4 replicates each. For each sampling time, values following the same letter were not significantly different according to Tukey-b test (α = 0.05).

Priming maritime pine megagametophytes with high temperatures induced constitutive changes in the ABA content of the plants derived by somatic embryogenesis from these explants. Basal levels of ABA showed significant differences among groups of plants (*p* = 0.035), and were higher in P50 than in NP and P37 at T0 (346.9 ± 29.1 front to 186.4 ± 14.3 and 122.9 ± 2.5 ng/g FW, respectively; Supplementary Table S1). ABA levels were also significantly affected by hydric stress (*p* < 0.001), and this variation along sampling times depended on the group of plants (Figure 5). As expected, the ABA concentration increased in needles of maritime pine plants during the drought period (T15 and T30 samples) up to 6.4-fold, and initial levels were recovered after 10 days in standard watering conditions. However, the ABA increase at T15 was lower in P50 plants than in NP and P37 plants (Figure 5).

### 2.5. Glutathione Metabolism in Maritime Pine Needles

**Figure 6:**
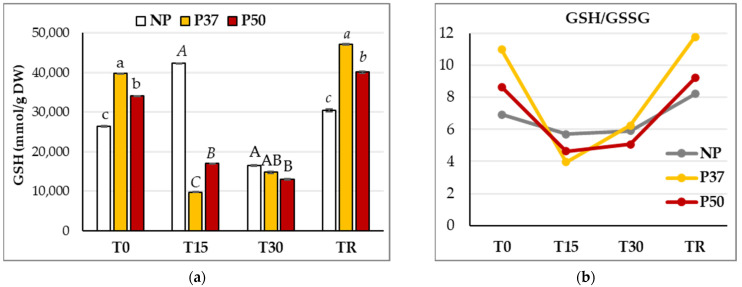
Reduced glutathione (mmol GSH/g DW) content (**a**), and ratio of GSH to oxidized glutathione (GSSG) (**b**) determined in needles of 3-year-old maritime pine plants generated by somatic embryogenesis from not-primed (NP) or primed at 37 °C (P37) or at 50 °C (P50) megagametophytes, when subjected to a 30-day hydric stress experiment. Data are mean ± SE of differences referred to mean initial GSH content, of needles taken during (T15), at the end (T30) of the experiment, and after a recovery period of 10 days (TR), with 3 replicates each. For each sampling time, GSH values following the same letter were not significantly different according to Tukey-b test (α = 0.05).

Reduced glutathione (GSH) content of maritime pine needles varied significantly during the hydric stress experiment (*p* < 0.001) and this variation depended on the group of plants. The initial GSH pool in NP plants was significantly (*p* < 0.001) lower than in primed plants, which showed also significant differences: 26,400 ± 681 μmol/g DW in NP plants front to 39,700 ± 734 and 34,047 ± 563 μmol/g DW in P37 and P50 plants, respectively (Supplementary Table S1). Higher basal contents of this compound were therefore detected in needles of P37 plants (Figure 6a), which showed a drastic decrease after 15 days without watering. Primed plants from the P50 group also showed this reduction in the GSH content of T15 samples, which contrasted with the significant increase determined in NP plants at this sampling time. At the end of the drought period (T30), plants showed similar GSH rates, since significant differences were observed only between NP and P50 plants. The GSH content of needles increased after the recovery period. Furthermore, irrespective of the group of plants, GSH levels determined in TR samples were significantly higher than those of T0 samples. Primed plants showed again significantly higher GSH rates than NP plants (Figure 6a).

As a result of this variation, significantly (*p* < 0.001) higher basal GSH/GSSG ratios were observed in needles from primed plants, particularly in P37 samples (11.0 ± 0.5), which also differed from ratios estimated in needles of P50 plants (8.6 ± 0.4), and the lowest values were found in NP plants (6.9 ± 0.4). Irrespective of the group of plants, the GSH/GSSG ratio also varied significantly (*p* < 0.001) during the drought experiment (Figure 6b). After 15 days without watering, the ratio between reduced and oxidized glutathione decreased, especially in samples from primed plants. In contrast, after the 30 days of drought, the GSH/GSSG ratio slightly increased; even initial levels were regained in NP plants. This trend was maintained after the recovery period; therefore, initial levels of the GSH/GSSG ratio were regained in primed plants, while in NP plants they were significantly higher than those determined initially (8.2 ± 0.5). Despite this increase, at TR, the GSH/GSSG ratio in NP plants was again significantly lower than in primed plants (*p* < 0.001).

### 2.6. Gene Expression Analyses

We investigated the expression in maritime pine needles of five genes coding for enzymes involved in cell response to stress: *Ascorbate Peroxidase* (*APX*), a precursor of the Cu-Zn-superoxide dismutase (*SOD*), and a *Caffeoyl-CoA O-Methyltransferase* (*CCOMT*), as well as a small *Heat Shock Protein70* (*HSP70*) and a putative *WRKY11* transcription factor (*WRKY*). Expression of these genes was analyzed previously [30] in the maritime pine primed embryogenic lines and in the derived somatic plants when subjected to a heat stress experiment. We also analyzed here three genes (*DNAJ, GST,* and *RD22*) reported as being involved in the stress response of drought-tolerant *P. pinaster* individuals [20]. Finally, we analyzed the expression levels of four *P. pinaster* dehydrin genes (*DHN1, DHN2, DHN3,* and *DHN4*) referred to be induced under drought stress [18]. Expression levels of these genes significantly varied along the hydric stress treatment, and differential expression was also observed among groups of plants for most of the cases. Basal expression levels (T0) of all these genes are shown in Supplementary Table S1.

**Figure 7:**
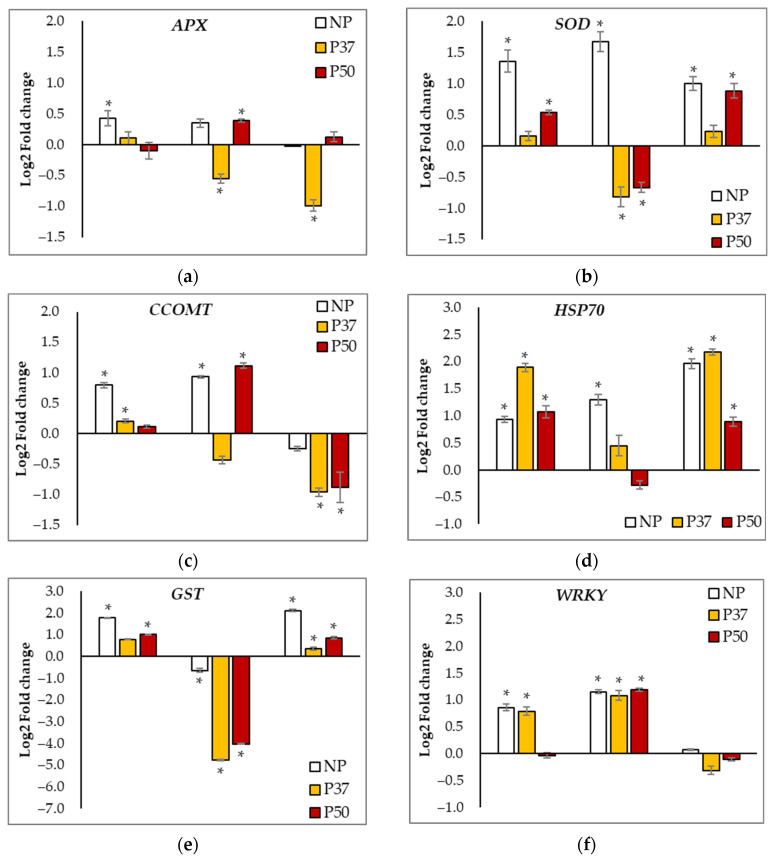

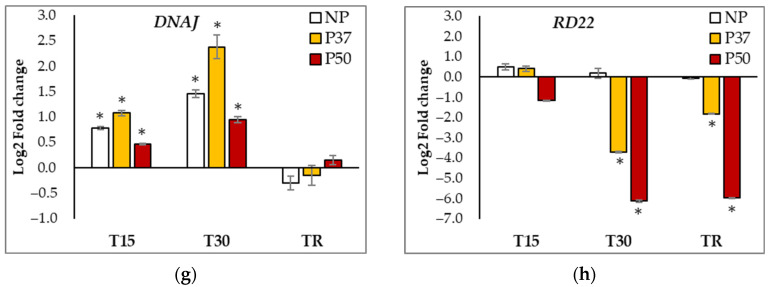
Relative gene expression (Log2 of fold change) at 3 sampling times (T15, T30, and TR) referred to the initial expression, of 8 genes [*APX* (**a**), *SOD* (**b**)*, CCOMT* (**c**), *HSP70* (**d**)*, GST* (**e**)*, WRKY* (**f**), *DNAJ* (**g**), and *RD22* (**h**)] in needles of 3-year-old maritime pine plants generated by somatic embryogenesis from not-primed (NP) or primed at 37 °C (P37) or at 50 °C (P50) megagametophytes, when subjected to a 30-day hydric stress treatment, and allowed to recovery for 10 days. HIS3 was used as a reference gene. Data are mean ± SE of 3 replicates; * denotes significant differential expression (*t*-test, α = 0.05).

Basal levels (T0 samples) of expression of the *APX* gene were higher in primed maritime pine plants than in NP plants; although, differences were significant only for P37 plants (0.175 ± 0.016 front to 0.131 ± 0.012, *p* = 0.008). Variations in *APX* gene expression along the hydric stress experiment depended on the group of plants, since, on average, it was significantly increased in NP plants (*p* = 0.013) and reduced in P37 plants (*p* = 0.020), while in P50 plants remained unchanged (*p* = 0.132). Furthermore, the pattern of variation in *APX* gene expression relative to initial levels significantly differed among groups of plants (Figure 7a).

Initial expression levels of the *SOD* gene were also significantly higher in primed than in NP plants (0.161 ± 0.006 in P37 and 0.187 ± 0.001 in P50 plants front to 0.061 ± 0.005 in NP plants; *p* < 0.001). Interestingly, hydric stress conditions induced opposite trends of variation in the expression of this gene, which significantly increased in NP plants while decreased in P37 and P50 plants (Figure 7b). Basal levels of *SOD* expression were regained after the recovery time in P37 plants, but increased significantly in NP and P50 plants. Expression of *APX* and *SOD* genes were inversely correlated, since we estimated a significant (*p* = 0.023) Spearman coefficient, ρ = −0.377 (Table 1).

Regarding the expression of the *CCOMT* gene, initial levels were similar in the three groups of plants (on average 0.086 ± 0.006), but were differentially affected by hydric stress. At the end of the drought period (T30 samples), gene expression increased in NP and P50 plants, while decreasing in P37 plants (Figure 7c). After the recovery period, *CCOMT* expression levels remained down-regulated, particularly in P37 plants as compared to NP plants.

Basal levels of expression of the *HSP70* gene also varied among groups of maritime pine plants (*p* = 0.039), since initially, the expression rate was significantly higher in P50 plants than in P37 plants (0.304 ± 0.017 front to 0.208 ± 0.016, respectively), while NP plants showed intermediate levels (0.248 ± 0.027). Drought conditions affected differentially gene expression, which increased at T15, particularly in P37 plants, but dropped at the end of the stress (T30) in primed plants while in NP plants, upregulation was maintained during the whole experiment (Figure 7d). When water supply was restored, *HSP70* expression increased, particularly in NP and P37 plants, while, in P50 plants, it increased at a lower extent.

A similar differential expression pattern among the three groups of plants along the drought experiment was observed for the *GST* gene (Figure 7e). In fact, the expression of *GST* correlated positively with that of the *HSP70* gene (ρ = 0.498, *p* = 0.002). Basal levels of *GST* expression were significantly lower in P37 plants as compared to NP and P50 plants (0.124 ± 0.024 front to 0.195 ± 0.010 and 0.212 ± 0.032, respectively; *p* = 0.010), and after 15 days under hydric stress this gene was upregulated, while expression decreased at the end of the stress (T30 samples), particularly in primed plants. After the recovery period, the GST gene was upregulated in maritime pine needles, particularly in those from NP plants.

Regarding the expression of the *WRKY* gene, basal levels also varied significantly among the three groups of plants, (*p* < 0.001), since the lowest rates were determined in NP plants (0.018 ± 0.001) as compared to those found in needles sampled from primed plants, which showed also significant differences (0.021 ± 0.001 in P37 and 0.027 ± 0.001 in P50 plants). Hydric stress induced higher transcription rates in T15 (NP and P37 plants) and in T30 samples (Figure 7f), while expression levels decreased after the recovery time and initial levels were recovered in P37 plants (*p* = 0.053) and almost regained in NP (*p* = 0.047) and P50 plants (*p* = 0.048). A similar pattern of variation along sampling times was observed for the expression of the DNAJ gene (Figure 7g), which was significantly altered by hydric stress (*p* < 0.001), but no significant differences were observed among the three groups of plants studied (*p* = 0.453). Furthermore, throughout our hydric stress experiment, expression in maritime pine plants of the transcription factor WRKY correlated positively with that of the CCOMT and DNAJ genes (ρ = 0.665 and ρ = 0.896, respectively; *p* < 0.001) and negatively with GST expression. Inverse correlations were also estimated between GST and APX and DNAJ expression levels (Table 1).

A priming-induced differential expression pattern among the three groups of plants was most evident for the *RD22* gene, which encodes a dehydration-responsive protein [20]. Transcription was never detected in NP plants, while basal levels observed in needles from primed maritime pine plants were 0.209 ± 0.002 and 0.268 ± 0.009 for P37 and P50 plants, respectively. Hydric stress induced down-regulation of this gene (Figure 7h) and initial expression rates were not regained after the recovery time. The expression profile of this gene was positively correlated with that of *APX* and *SOD* genes (Table 1).

**Figure 8:**
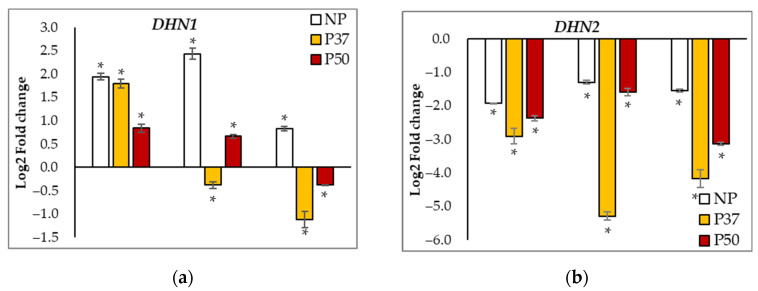

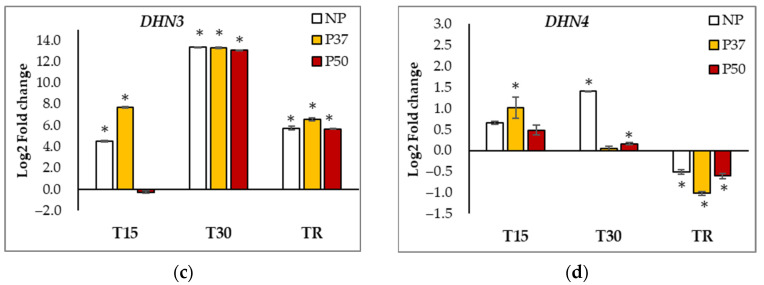
Relative gene expression (Log2 of fold change) at 3 sampling times (T15, T30, and TR) referred to initial expression of 4 dehydrin genes [*DHN1* (**a**), *DHN2* (**b**), *DHN3* (**c**), and *DHN4* (**d**)] in needles of 3-year-old maritime pine plants generated by somatic embryogenesis from not-primed (NP) or primed at 37 °C (P37) or at 50 °C (P50) megagametophytes, when subjected to a 30-day hydric stress treatment, and allowed to recover for further 10 days. *HIS3* was used as reference gene. Data are mean ± SE of 3 replicates; * denotes significant differential expression (*t*-test, α = 0.05).

In our 3-year-old somatic embryogenesis-derived plants, significant differences were found in basal levels of expression of *DHN1* and *DHN2* genes (*p* = 0.021 and *p* = 0.027, respectively), since at T0 expression of these genes was higher in P50 plants than in NP plants, while P37 plants showed intermediate values (Supplementary Table S1). After 15 days without watering, *DHN1* expression increased in the three groups of plants (Figure 8a), but at the end of the drought period (T30 samples), expression of this gene remained high in NP and P50 plants, while decreased in P37 plants, and stress-induced altered levels were maintained after the recovery period. Expression of the *DHN2* gene was significantly down-regulated under drought conditions in the three groups of plants, particularly in P37 plants, and initial levels were not regained after the recovery time (Figure 8b).

Expression of the *DHN2* gene inversely correlated with that of *HSP70* (ρ = −0.480, *p* < 0.001) and *DHN3* (ρ = −0.540, *p* = 0.001) genes. Irrespective of the group of maritime pine plants, *DHN3* expression strongly depended on drought stress conditions (*p* < 0.001), since we estimated 2^−ΔCT^ rates <0.002 in T0, T15, and TR samples, which contrasted with the peak of expression observed in T30 samples. Although the observed pattern of variation was similar for the three groups of plants (Figure 8c), it is interesting to note that *DHN3* expression levels at T30 were significantly higher in primed plants, since we determined expression rates of 0.030 ± 0.001, 0.088 ± 0.007, and 0.171 ± 0.002, for NP, P37, and P50 plants, respectively. In addition, expression of the *DHN3* gene correlated positively to that of *DNAJ* and *WRKY* genes, and negatively with that of the *GST* gene (Table 1).

A different expression profile was observed for the *DHN4* gene, which was upregulated under hydric stress conditions in NP plants while in primed plants the increase was significant only in P50 plants after 30 days (Figure 8d). When watering was restored, *DHN4* expression rates decreased below initial levels, which were significantly higher in P50 plants (0.177 ± 0.006) than in NP (0.110 ± 0.002) or P37 (0.129 ± 0.022) plants. Expression of this gene was significantly and positively correlated to that of *DHN1* (ρ = 0.743, *p* < 0.001). Furthermore, *DHN1* and *DHN4* expression levels were positively correlated with those of *CCOMT, DNAJ,* and *WRKY* genes (Table 1).

## 3. Discussion

In a previous work, we demonstrated that heat priming during somatic embryogenesis improved maritime pine adaptation to heat stress [30]. In this work, our goal was to test whether this priming would induce cross-tolerance to subsequent mild drought stress. To this end, we analyzed the expression of some of the genes and metabolites that protect cells for changes in osmotic potential and oxidative stress involved in conifer response to drought stress. The recovery after stress might indicate the capacity of the plants to return to the undisturbed ecosystem state and is related to their resilience, which depends on the scale and complexity of the function examined [34]. Since, in conifers, slow recovery has been associated with higher mortality risk [35], we measured the extent of the maritime pine recovery ten days after irrigation was restored.

Plants growing for 3 years in the greenhouse displayed constitutive physiological differences, since, at T0, needles from heat-priming-derived plants showed higher starch, total protein content, and GSH levels than those from NP control plants. Furthermore, the transcription factor *WRKY*, the *ROS* scavenging *SOD,* and the dehydration-responsive *RD22* genes were upregulated in both P37 and P50 plants (Supplementary Table S1). Moreover, different profiles were found when comparing P37 with P50 plants: priming at a lower temperature (37 °C) favored proline accumulation and increased ΦPSII yield, upregulated *APX,* and decreased *GST* gene expression. In turn, priming at higher temperatures (50 °C) produced ABA accumulation, and increased expression of dehydrin genes *DHN1, DHN2,* and *DHN4*.

Maritime pine is considered a drought-avoiding species as it displays some mechanisms such as sensitive stomata and rapid osmotic adjustment in response to this stress [in 21]. ABA is involved in stomatal closure, shoot growth, and water uptake, and molecular studies revealed that it also regulates many structural genes in conifers [6,36]. To regulate osmotic adjustment and keep turgor pressure, plants accumulate proline, starch, and soluble sugars, which contribute to the acquisition of desiccation tolerance in tree species [18,37,38]. Starch degradation provides energy and carbon when photosynthesis may be potentially limited; therefore, it has been correlated with stress tolerance. In fact, strategies for increasing carbohydrate storage are known to increase drought resistance in seedlings and can also be seen as an acclimation induced by priming [34]. However, several studies also reported an increase in starch accumulation under stress [36,39].

In our experiments, after 30 days without irrigation and irrespective of the plant origin (primed or not), the water content in needles dropped about 15%, in spite of the proline and TSS accumulation, but plants did not show a visible drought phenotype except for the above-mentioned epinasty in basal needles. We suggest that the observed ABA accumulation (Figure 5) induced stomata closure, decreasing photosynthesis (ΦPSII yield, Figure 2), which, in turn, induced starch degradation to produce TSS accumulation and, then, maintained needle turgor and metabolic processes. Interestingly, P37 and P50 plants activated starch degradation to produce TSS accumulation earlier (15 days) than NP plants, which, at this time, increased RWC and starch accumulation (Figure 3a,b). Similar results for the starch level in P50 maritime pine plants were obtained for *Eucalyptus globulus* subjected to drought and 40 °C [40].

The reduction in water content (RWC) of maritime pine needles observed at the end of the drought treatment was similar to that reported for *P. massoniana* seedlings by [37] in a drought treatment characterized by a 50% reduction in hydraulic conductivity. Moreover, RWC decreases did not differ among primed and control (NP) plants; results that agree with those reported by [29] for one-year-old *P. radiata* plants. Note, however, that the RWC of primed maritime pine plants was recovered faster than in controls (Figure 1a). These results contrasted with those observed in our previous work after applying heat stress [30], when RWC and proline increased in both control and primed maritime pine plants. Furthermore, in this experiment, NP plants showed the highest proline content [30]. Differences in the maritime pine response to heat and drought stresses were also observed for photosynthesis parameters, since heat did not affect ΦPSII but rather induced higher levels of chlorophyll b and carotenoids in 2-year-old plants [30], results which contrasted with those reported here in 3-year-old maritime pines under hydric stress, when ΦPSII was significantly reduced, and pigment contents remained unaffected. According to [21], ΦPSII damage varies depending on the species and the types of stress to which the plants are subjected. In fact, in citrus plants, combination heat and drought stresses dealt more damage to ΦPSII than just drought stress [41], while in *Eucalyptus globulus,* the combination of both damaged ΦPSII less than only drought [40].

Dehydrin proteins (DHN) accumulate to relatively higher amounts in multiple tissues during drought and associated stresses, performing osmotic adjustment by sequestering ions, stabilizing membranes, and/or by functioning as chaperones. Their expression has been associated with ABA, which confers a better response to abiotic stresses [42,43,44]. The high content of this hormone in P50 plants might account for the upregulation in three *DHN* genes that was not observed in NP and P37 plants. Early up-regulation of several transcription factors has been observed under water deficit in conifers, but [20] referred that *P. pinaster* tolerant individuals constitutively expressed stress-related genes, while in sensitive individuals, these genes are induced by the onset of stress. In our study, the transcription factor *WRKY* and the dehydration-responsive *RD22* genes were upregulated in primed plants (Supplementary Table S1), being some members of the *WRKY* transcription factors family described as priming marker genes associated with epigenetic modifications [45]. *WRKY* plays a crucial role in monitoring plant responses to various abiotic stimuli by coordinating intrinsic signals related to developmental processes and by interacting with other proteins in the plant signaling network [46]. In fact, higher constitutive expression levels of this gene were already reported in 2-year-old maritime pine P50 plants and in the embryogenic lines after priming [30]. The higher expression rates of the *WRKY* gene could therefore indicate a pre-adaptive tolerance response of these plants. The *RD22* gene is a molecular link between ABA signaling and abiotic stress responses [47,48]. Messengers for the RD22 protein were not found in samples from control plants, while, in primed plants, the gene was highly expressed at T0, but expression decreased during the drought experiment and was not recovered after restoring the water supply (Figure 7h). It is worth noting that the constitutive expression of the *RD22* gene in *P. pinaster* has been associated with drought tolerance genotypes [20]. Chaperonins such as HSP and DNAJ proteins are also synthesized by plants to reduce cellular damage. The DNAJ co-chaperones recognize unfolded substrates and deliver them to HSP70 chaperones while stabilizing their interaction with the substrate, thus controlling protein homeostasis [49]. Furthermore, assessment of the *DNAJ* gene expression allowed to discriminate drought tolerant cultivars in maize [50]. In our experiment, the expression of both *HSP70* and *DNAJ* increased during drought stress. Interestingly, *DNAJ* and *WRKY* expression patterns were significantly correlated, and the expression of both genes showed also a significant correlation with that of three dehydrin-coding genes: *DHN1, DHN3,* and *DHN4*. These genes were upregulated under water deficit, while *DHN2* expression decreased drastically during the experiment and was not recovered when watering was restored (Figure 8).

Exposure of plants to osmotic stress led to oxidative events in cells, which induces numerous genes that code for antioxidant enzymes such as superoxide dismutase (SOD), ascorbate peroxidase (*APX*), catalase, and glutathione reductase (GR). For example, in *P. halepensis,* drought increased the expression of *GR* and *CuZnSOD* [51]. Moreover, *CCOMT* has been found to play a positive role in the response to drought stress in arabidopsis, by regulating H_2_O_2_ accumulation and ABA signaling [52]. Glutathione S-transferases are a superfamily of enzymes that quench reactive molecules with the addition of glutathione and protect cells from oxidative damage, metabolizing the toxic products of lipid peroxidation, damaged DNA, and other molecules [53,54]. In this work, we found that the initial expression levels of the ROS scavenging *SOD* and *APX* were higher in primed than in control maritime pine plants, while drought stress affected differentially the three groups of plants, as occurred for the *CCOMT* and *GST* genes. Early response to the stress of maritime pine plants included up-regulation of these four genes, but this effect was more pronounced in NP plants. In many species, it has been observed that the overexpression of certain genes, such as *SOD* and *APX* [55], confers a cross-resistance to different abiotic stresses (thermal, osmotic, oxidative, and others), and increasing ROS scavenging enzymes is associated with acquired stress memory [56]. Then, our results suggest that the priming technique in pine can be a suitable system to obtain this cross-resistance via the increase in the level of expression of these genes (Figure 7).

Finally, we analyzed the needle’s contents in reduced glutathione (GSH) and glutathione disulfide (GSSG). Higher GSH levels and GSH/GSSG ratios, as well as enhanced SOD, catalase, GPX, and GST activities, have been correlated with salt tolerance in several plants [57]. Initial GSH contents of primed maritime pine plants were significantly higher than those from control plants, but, after 15 days without watering, GSH contents increased 1.6-fold in NP plants while decreasing 4-fold in P37 and 2-fold in P50 plants. Although the GSH/GSSG ratios showed significant decreases, particularly in primed plants, during the water deficit period, they were recovered after restoring the water supply (Figure 6). The major recovery of this ratio in primed plants was also observed in the roots of maize seedlings subjected to heat shock and then to aluminum stress, compared to maize seedlings subjected to only one of these stresses, which unequivocally suggests that GSH is a key regulator involving in heat shock priming-induced cross-tolerance to Al stress in wheat plants [56,58].

When all these results are considered, we can conclude that applying high temperature pulses during the induction of somatic embryogenesis in maritime pine megagametophytes produces epigenetic marks in the derived somatic plants that resulted in transcriptomic and physiological changes that can help in cross-tolerance to drought stress. These changes consist of constitutive higher contents of needles in osmoprotectant compounds such as proline, soluble proteins, GSH, and starch, as well as higher expression levels of genes coding for transcription factors involved in early stress response (*WRKY, RD22*), of genes coding for proteins known to avoid cell damage (HSP70, DHN1, DHN2, and DHN4), and of genes coding for enzymes involved in an oxidative stress response (*APX, SOD,* and *GST*). After 15 days of water withdrawal, P37 plants accumulated more proline, TSS, and dehydrins than NP plants, indicating their primed state. Finally, P37 and P50 plants recovered faster from stress in terms of RWC, TSS, and GSH, indicating that any of the priming treatments is suitable to induce a faster response to mild-short-term drought stress. These plants are now under field conditions and results presented here should be validated by incorporating long-term droughts on larger trees, where parameters such as hydraulic traits and secondary metabolites accumulation will be also studied.

## 4. Materials and Methods

### 4.1. Plant Material and Drought Experiment Performed in Greenhouse

**Figure 9:**
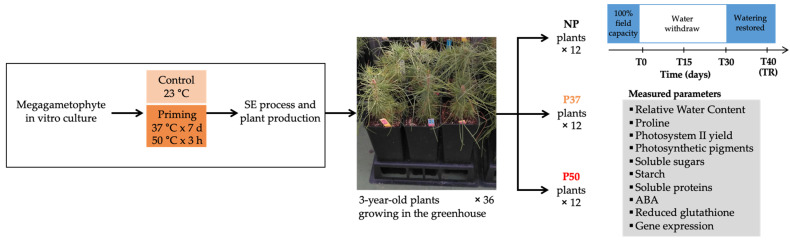
Experimental design. Maritime pine plants were obtained following the somatic embryogenesis protocol described in [30]. Not-primed (NP) and primed (P37 and P50) plants were maintained for 3 years in a greenhouse. The drought experiment was performed using 12 plants from each group, which were first watered up to field capacity, and then not watered for 30 days after which watering was restored. Samples were collected 24 h after being watered up to field capacity (T0), after 15 days (T15) and 30 days (T30) of not watering, and 10 days after watering was restored (TR). Samples were collected at these times and used to measure the different parameters.

Maritime pine (*Pinus pinaster*) plants were obtained following the somatic embryogenesis protocol developed by our group [32] under standard (23 °C) or priming conditions (37 °C for 7 days or 50 °C for 3 h) as described in [30]. Then, not-primed (NP) and primed (P37 and P50) plants were cultivated in 2 L pots containing a mixture of peat moss and perlite 7:3 for 3 years in a greenhouse (SCSIE, University of Valencia) at 24 ± 4 °C with a 16 h photoperiod and 200–300 W·m^−2^ irradiance and weekly watered and fertilized. The drought experiment was performed using at least 12 plants (35–40 cm in length) from each group, which were first watered up to field capacity, and allowed to stand for 24 h before sampling mature needles, which were named T0 samples. Plants were not watered for the next 30 days, and needles were collected after 15 days (T15 samples) and at the end of the hydric stress treatment (T30 samples). Then, watering was restored, and needles were sampled after 10 days recovery in standard cultivation conditions (TR samples). All these samples were frozen in liquid nitrogen, and stored at −80 °C until analyzing. The experimental design is depicted in Figure 9.

### 4.2. Physiological Characterization of 3-Year-Old Maritime Plants Subjected to Drought Stress

Relative water content (RWC) of maritime pine needles was determined following the protocol described by Escandón et al. [59], using 3 fragments (1 cm long) in each six replicates per sampling point. Samples fresh weight (FW) was registered, and needles were maintained with de-ionized water for 24 h in dark at 4 °C, after which turgid weight (TW) was recorded. Then, needles were dried at 80 °C for 72 h, and dry weight (DW) was registered. RWC was calculated as described in Pérez-Oliver et al. [30] by using the following Equation (1):

RWC (%) = (FW − DW)/(TW − DW) × 100(1)

Proline was quantified in needles according to Bates et al. [60] with modifications, in six replicates per treatment. About 100 mg of frozen needles was homogenized in 3% sulfosalicylic acid (5 μL/mg FW), and centrifuged (13,000 rpm for 5 min). A mixture with 100 μL of 3% sulfosalicylic acid, 200 μL of glacial acetic acid, and 200 μL of acidic ninhydrin was added to 100 μL of the supernatant of the extract, and the resulting mixture was vortexed and incubated at 96 °C for 1 h. After that, the reaction was finished on ice for 10 min. Samples were extracted in 1 mL of toluene and vortexed for 20 s, and the formation of two phases was observed. The absorbance of the chromophore-containing toluene phase was read at 520 nm (Eppendorf BioSpectrometer^®^ basic; Sigma-Aldrich®, Merck KGaA, Darmstadt, Germany) using toluene as blank reagent, and proline concentration was determined from a Sigma-Aldrich^®^ L-proline standard curve with 6 points (0–150 μg/mL).

To estimate photosynthetic activity in maritime pine plants growing at the greenhouse, the photosystem II operating efficiency (ΦPSII) was analyzed in needles using a pulse-amplitude modulation fluorimeter (MINI PAM; Walz, Effeltrich, Germany), according to Nebauer et al. [61]. Two measurements of five needles in the mid part of the plant were performed; therefore, a total of 10 replicates were determined for each group of plants, treatment, and sampling period. Needles were pre-adapted in the dark for 20 min, and then exposed to a light flash, taking one measure in darkness and another in light, both at a wavelength of 350–400 nm. Estimates of ΦPSII were obtained by measuring variable fluorescence (Fv), and calculating the difference (Fv = Fm − F0) between the maximum fluorescence (Fm), after the light flash, and the minimum fluorescence (F0), in the absence of light. The estimated ΦPSII represents the proportion of the energy absorbed by the chlorophyll of PSII that is being used to drive the photochemical process; therefore, it is a measure of the efficiency of linear electron transport.

Chlorophyll and carotenoids were extracted and analyzed in needles of maritime pine plants according to Lichtenthaler [62] with some modifications, in 6 replicates per treatment. A total of 300 mg of needles were ground in metal containers with a metal sphere (50 mm ø) using the MM 400-RetschTM mixer for 30 s. After grinding, three aliquots of 100 mg each were prepared and pigments were extracted in 10 mL of 100% (*v/v*) acetone and centrifugated (10,000 rpm, 10 min, 4 °C). After that, absorbances of the supernatants were read at 470, 645, and 662 nm (Eppendorf BioSpectrometer^®^ basic), and the concentration of each pigment was determined.

Total soluble sugars (TSS) and starch contents were determined in needles of maritime pine plants as described by Rodríguez et al. [63], in 6 replicates per treatment. Extracts were obtained from 50 mg of frozen needles ground and homogenized in 10 mL of 80% (*v/v*) ethanol, by using a MM 400-RetschTM mixer for 40 s. The mixture was incubated at 80 °C for 1 h and centrifuged (6000 rpm, 20 min, 4 °C). After that, 2.5 mL of a solution of Sigma-Aldrich^®^ anthrone (0.25 g of anthrone in 100 mL of 95% sulfuric acid) was added to 1 mL of the supernatant, and the mixture was vortexed and incubated at 100 °C for 15 min. After cooling down, the absorbance was read at 620 nm. Starch content was determined from the pellet resulting from the centrifugation of the initial extract, which was incubated in 10 mL of 30% (*v/v*) perchloric acid for 16 h at room temperature. After centrifugation, 1 mL of the supernatant was mixed with 2.5 mL of anthrone, vortexed, and incubated at 100 °C for 15 min. The mixture was cooled down and the absorbance was read at 620 nm. Both TSS and starch contents were calculated against a Sigma-Aldrich^®^ D-glucose standard curve (0–800 μM), using anthrone as a blank.

Total protein content was determined colorimetrically using the Bradford dye-binding assay with bovine serum albumin (BSA, Sigma-Aldrich^®^) as reference. For this analysis, about 30 mg of frozen needles was ground and homogenized in 1 mL of PBS buffer 1x (NaCl 140 mM, Na_2_HPO_4_·7H_2_O 8 mM, KH_2_PO_4_ 2 mM, KCl 10 mM, pH 7.4 with HCl), by using a MM 400-RetschTM mixer for 1 min. The extract was maintained on ice for 30 min and then centrifuged (15,000 rpm, 20 min, 4 °C). For the assay, a mixture of 10 μL of supernatant and 180 μL of Bradford solution (diluted 1:8) was mixed in a microplate, shacked briefly, and incubated on ice for 10 min. The absorbance was read at 620 nm, and the total protein content was calculated against a BSA standard curve (0–0.25 mg/mL). Three biological replicates were prepared for each treatment.

Concentration levels of ABA were determined according to a modified method based on that described by Šimura et al. [64]. Briefly, samples containing 10 mg fresh weight were extracted in aqueous solution of 50% acetonitrile (*v/v*). Crude extracts were loaded onto conditioned Oasis HLB columns (30 mg/mL, Waters) and washed with aqueous solution of 30% acetonitrile (*v/v*). Flow-through fractions containing purified analytes were collected and evaporated to dryness in vacuum evaporator. Samples were dissolved in 30 μL of mobile phase and then analyzed using an Acquity I-class system (Waters, Milford, MA, USA) combined with a triple quadrupole mass spectrometer (Xevo TQ-S, Waters). A mixture of stable isotope-labeled standards of hormones was added to validate the LC-MS/MS method, and concentration levels were calculated using the isotope dilution method. All data were processed with Mass-Lynx V4.2 software (Waters).

Reduced glutathione (GSH) and glutathione disulfide (GSSG) were quantified in needles according to Rosa-Téllez et al. [65] with some modifications. Extraction PBS buffer (100 μL) and 2 μL of a 100 mM solution of N-Ethylmaleimide were used to homogenize 50 mg of frozen needles in a 1.5 mL tube. The mixture was vortexed and centrifugated (13,000 rpm, 10 min, 4 °C). The supernatant was collected in a new tube and centrifugated again. After that, 18 μL of cold perchloric acid (8%) was added to 80 μL of the supernatant and the mixture was shacked manually and centrifugated (13,000 rpm, 10 min, 4 °C). The supernatant was collected and directly used for GSH/GSSG quantification with a triple quadrupole mass spectrometer (ACQUITY^®^ TQD, Waters) equipped with a Z-spray electrospray ionization source and a C8 Kinetex column (2.1 × 100 mm, 1.7 μm; Phenomenex, USA). Three biological replicates were prepared for each treatment.

### 4.3. Gene Expression Analyses

Total RNA was isolated from frozen samples of needles sampled in greenhouse-growing plants following the protocol described by Canales et al. [66]. Genomic DNA was degraded by using the Recombinant DNase I (RNase-free, Takara Bio Inc., Shiga, Japan), following manufacturer’s instructions. RNA quantity and quality were assessed by a NanoDropTM (Thermo Fisher Scientific, Waltham, MA, USA). Synthesis of cDNA was performed by the PrimeScriptTM RT Reagent Kit (Perfect Real Time, Takara Bio Inc.), following manufacturer’s instructions. Real-time PCR amplifications were performed in a StepOne Plus (Applied Biosystems, Foster City, CA, USA), using a final volume of 20 µL containing 0.3 µM of each primer and 10 µL of SYBR Green I Master mix (Takara Bio Inc.) in triplicate for each sample. Amplification conditions were 10 min × 95 °C, and 40 cycles of 15 s × 95 °C and 60 s × 55 °C. Gene coding for Histone 3 was used as a reference to estimate expression levels (2^(−ΔCt)^ method). Differential expression rates at T15, T30, and TR for each group of plants were estimated as the base 2 logarithm of the ratio between gene expression at each sampling time and that at T0. Analyzed genes (12 in total) are summarized in Table 2; primer details are described in Supplementary Table S2. Three biological replicates were prepared for each treatment.

### 4.4. Statistical Analyses

Data recorded in the different experiments were subjected to analysis of variance using the SPSS software v.25 (IBM Statistics). When appropriate, Tukey-b or Tamhane mean separation tests were also performed. When data did not adjust to a normal distribution (Kolmogorov–Smirnoff test), significant differences were assessed using the Kruskal–Wallis non-parametric ANOVA test.

## Figures and Tables

**Table 1:** Coefficient of correlation (Spearman’s ρ) among expression levels of 12 genes in needles of 3-year-old maritime pine somatic plants derived from embryogenic lines not primed or primed with high temperatures, when they were subjected to hydric stress for 30 days and allowed to recovery during 10 days.

	*APX*	*SOD*	*CCOMT*	*HSP70*	*GST*	*WRKY*	*DNAJ*	*RD22*	*DHN1*	*DHN2*	*DHN3*
*SOD*	−0.377 *										
*CCOMT*	0.127	−0.105									
*HSP70*	−0.290	0.445 **	−0.317								
*GST*	−0.541 **	0.321	−0.288	0.498 **							
*WRKY*	0.250	−0.050	0.665 **	−0.237	−0.407 *						
*DNAJ*	0.031	0.022	0.526 **	−0.202	−0.396 *	0.896 **					
*RD22*	0.387 *	0.387 *	−0.076	−0.037	−0.137	0.007	−0.036				
*DHN1*	−0.305	0.277	0.697 **	0.244	0.283	0.551 **	0.467 **	−0.135			
*DHN2*	−0.019	−0.069	0.328	−0.480 **	−0.026	−0.120	−0.184	0.138	0.120		
*DHN3*	0.274	−0.119	0.238	0.138	−0.502 **	0.658 **	0.603 **	−0.190	0.201	−0.540 **	
*DHN4*	−0.042	0.310	0.675 **	−0.090	−0.095	0.675 **	0.661 **	0.289	0.743 **	0.297	0.130

* *p* < 0.05; ** *p* < 0.01.

**Table 2:** *Pinus pinaster* genes whose expression was analyzed in plant needles.

Gene	Gene Annotation	Reference
*APX*	*Ascorbate Peroxidase*	[30]
*CCOMT*	*Caffeoyl-CoA O-Methyltransferase*	
*HSP70*	*Heat Shock Protein 70 (HSP70)*	
*SOD*	*Cu-Zn-superoxide dismutase precursor*	
*WRKY*	*Transcription factor WRKY11*	
*DNAJ*	*Chaperone protein dnaj chloroplastic-like*	[20]
*GST*	*Glutathione S-transferase*	
*RD22*	*Dehydration-responsive protein RD22*	
*DHN1*	*Ppindhn1*	[67]
*DHN2*	*Ppindhn2*	
*DHN3*	*Ppindhn3*	
*DHN4*	*Ppindhn4*	
*HIS3*	*Histone 3 (HIS3)*	[68]

## Data Availability

Data are included in the ms.

## Supplementary Materials

The following supporting information can be downloaded at: https://www.mdpi.com/article/10.3390/ijms24119299/s1.
