# Supplementary material for: Heat-Priming during Somatic Embryogenesis Increased Resilience to Drought Stress in the Generated Maritime Pine (Pinus pinaster) Plants
Source: Int J Mol Sci. 2023 May 26;24(11):9299. doi: 10.3390/ijms24119299 (PMC10253388; doi:10.3390/ijms24119299)
Supplement: Supplementary file 1 [file ijms-24-09299-s001.zip › ijms-2400011-supplementary.pdf]

Supplementary Materials

Table S1. Constitutive values (T0 samples) of the analysed parameters found in control (NP) and primed (P37 and P50) maritime pine plants.

	Group	NP	P37	P50
**Osmotic adjustment**	**RWC (%)**	80.2	80.8	82.8
	**Proline (µg/mg FW)**	18.1	22.0**	14.6
**Photosynthesis parameters**	**Yield PSII**	0.681	0.712*	0.678
	**Chlorophyll-a (mg/gFW)**	0.545	0.709	0.507
	**Chlorophyll-b (mg/gFW)**	0.148	0.334	0.143
	**Carotenoids (mg/gFW)**	0.136	0.155	0.133
**Basal metabolism**	**TSS (M/mg FW)**	7.7	6.5	8.0
	**Starch (M/mg FW)**	32.8	49.4*	49.5*
	**Protein (mg/g FW)**	0.67	2.34**	3.16**
**ABA content (ng/g FW)**		0.186	0.186	0.123
**GSH content (µmole/g DW)**		26.4	26.4	39.7**
**Gene expression ($2^{(-\Delta CT)}$)**	***APX***	0.131	0.175*	0.153
	SOD	0.060	0.161**	0.187**
	CCOMT	0.079	0.100*	0.080
	HSP70	0.248	0.208	0.304*
	GST	0.195	0.124*	0.212
	WRKY	0.018	0.021**	0.026**
	DNAJ	0.127	0.110	0.175*
	RD22	0.000	0.209*	0.268*
	DH1	0.047	0.058	0.078**
	DH2	0.009	0.019	0.021**
	DH3	0.000	0.000	0.000
	DH4	0.110	0.129	0.177*

*$p < 0.05$; ** $p < 0.01$ significant difference as compared with NP plants.

Table S2. Primers used for qPCR analyses.

Gene code	Protein	Primers		Reference
		Forward	Reverse	
APX	*Ascorbate peroxidase*	GGTCTGGACATCGCAGTTAG	CCACACCAGCCAACTGATAA	[1]
CCO	*Caffeoyl CoA O-methyltransferase*	GCCGATGAGGGTCAATTTCT	AATGCAAGGGCTGTGCT	
HSP70	*Heat shock protein HSP* 70	CACCAGCAGTGGGAATATCA	AGGGCATCTGCAATCCTATC	
SOD	*Cu-Zn-superoxide dismutase precursor*	CAATGGCTGCATGTCAACAG	CCATCAGAACCCGCAACTAT	
WRKY	Putative *WRKY* factor	CCCATTATCCTCCACTAACTCC	GCTGAGAGATCGAACCAGTATAG	
DH1	*PpinDhn1*	GGAGAAGAAGTTGGGTGTGCTTGG	TCCTCTTCCGCATCATTCTTCTGG	[2]
DH2	*PpinDhn2*	AAGCTGCCGGGACACCATAACA	CATGCATGCAACGCCCTAAA	
DH3	*PpinDhn3*	GGTGGGCACCAGGCTTCTTCA	TCATGGTGTCCAGGAAGCTTGTCC	
DH4	*PpinDhn4*	GCCTGAGAACATTGATGGCAATGATGA	GCGGATCCGTCGGCTCACA	
DNAJ	*Chaperone protein DnaJ chloroplast c-like*	ATGGATGGGATGGGAAGGAG	GCCCAACTATTTACAAGGCGAA	[3]
GST	*Glutathione S-transferase*	TCCCGGACCCTCTTAAGGTT	ATGGCTACGCTGATGCAAGT	
RD22	*Dehydration-responsive protein rd22*	CTCATCCCAGAAAACGTCGG	TACCAATACTTCCGGGGTGG	
HIS	*Histone* 3 (HISTO 3)	GCTGAGGCTTACCTTGTG	CCAGTTGTATATCCTTAGGCATAA	[4]

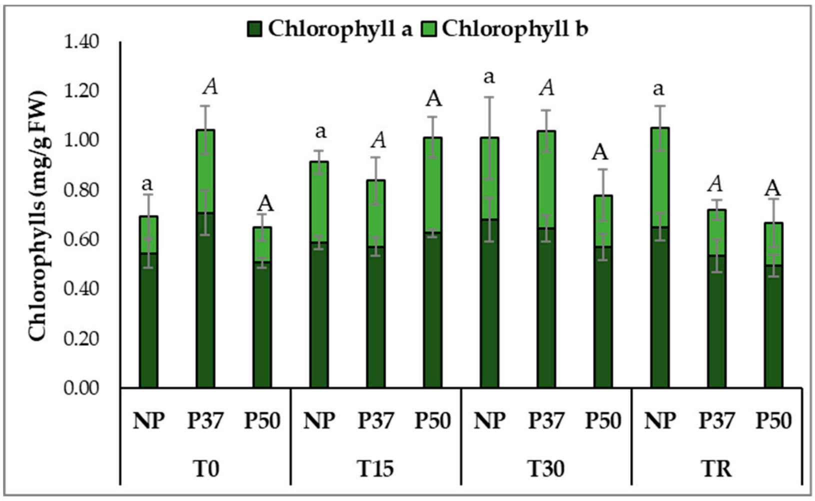

(**a**)

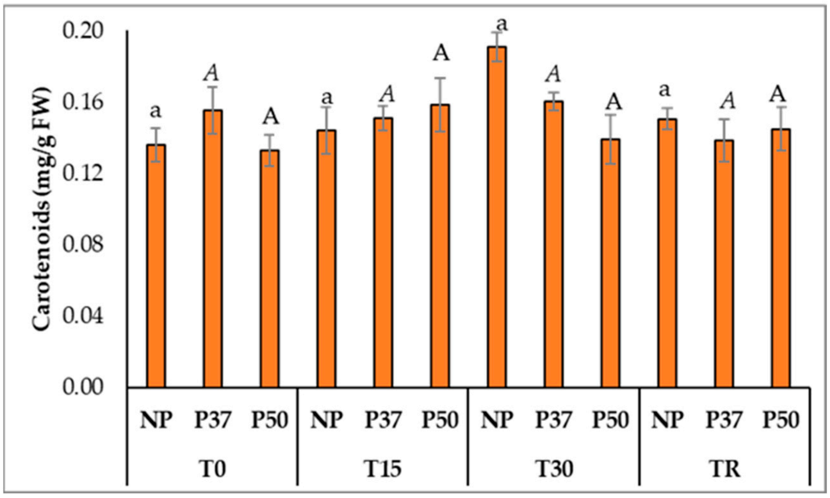

(**b**)

Figure S1. Chlorophyll a and chlorophyll b contents (**a**) and carotenoids content (**b**) in needles of 3-year-old maritime pine plants derived by somatic embryogenesis from not primed (NP) or primed at 37 °C (P37) or at 50 °C (P50) megagametophytes, when subjected to a 30-day hydric stress treatment and allowed to recovery for further 10 d. Data are mean ± SE of 3 replicates sampled before (T0), during (T15), at the end (T30), and after the recovery period (TR). For each group of plants, values followed of the same letter were not significantly different according to Tukey test (α = 0.05).

References

1. Pérez-Oliver, M.A.; Haro, J.G.; Pavlović, I.; Novák, O.; Segura, J.; Sales, E.; Arrillaga, I. Priming Maritime Pine Megagametophytes during Somatic Embryogenesis Improved Plant Adaptation to Heat Stress. *Plants* **2021**, *10*, 446, https://doi.org/10.3390/plants10030446.
2. Velasco-Conde, T.; Yakovlev, I.; Majada, J.P.; Aranda, I.; Johnsen, . Dehydrins in maritime pine (Pinus pinaster) and their expression related to drought stress response. *Tree Genet. Genomes* **2012**, *8*, 957–973, https://doi.org/10.1007/s11295-012-0476-9.
3. De María, N.; Guevara, M. Á.; Perdiguero, P.; Vélez, M.D.; Cabezas, J.A.; López-Hinojosa, M.; Li, Z.; Díaz, L.M.; Pizarro, A.; Mancha, J.A.; et al. Molecular study of drought response in the Mediterranean conifer Pinus pinaster Ait.: Differential transcriptomic profiling reveals constitutive water deficit-independent drought tolerance mechanisms. *Ecol. Evol.* **2020**, *10*, 9788–9807, https://doi.org/10.1002/ece3.6613.
4. De Vega-Bartol, J.J.; Santos, R.R.; Simões, M.; Miguel, C.M. Normalizing gene expression by quantitative PCR during somatic embryogenesis in two representative conifer species: Pinus pinaster and Picea abies. *Plant Cell Rep.* **2013**, *32*, 715–729, https://doi.org/10.1007/s00299-013-1407-4.
